# Comparison of two novel swept-source optical coherence tomography devices to a partial coherence interferometry-based biometer

**DOI:** 10.1038/s41598-021-93999-8

**Published:** 2021-07-21

**Authors:** Tommy C. Y. Chan, Marco C. Y. Yu, Vivian Chiu, Gilda Lai, Christopher K. S. Leung, Poemen P. M. Chan

**Affiliations:** 1 Department of Ophthalmology and Visual Sciences, The Chinese University of Hong Kong, Hong Kong Eye Hospital, Kowloon, Hong Kong, People’s Republic of China; 2 Department of Ophthalmology, Hong Kong Sanatorium & Hospital, Hong Kong, People’s Republic of China; 3 Singapore Eye Research Institute, Singapore National Eye Centre, Singapore, Singapore; 4 Hong Kong Eye Hospital, Hong Kong, SAR People’s Republic of China; 5 Department of Ophthalmology, The University of Hong Kong, Hong Kong, People’s Republic of China

**Keywords:** Corneal diseases, Refractive errors

## Abstract

To evaluate the repeatability and agreement of corneal and biometry measurements obtained with two swept-source optical coherence tomography (SSOCT) and a partial coherence interferometry-based device. This is a cross-sectional study. Forty-eight eyes of 48 patients had three consecutive measurements for ANTERION (Heidelberg Engineering, Germany), CASIAII (Tomey, Japan) and IOLMaster500 (Carl Zeiss Meditec, USA) on the same visit. Mean keratometry (Km), central corneal thickness (CCT), anterior chamber depth (ACD) and axial length (AL) were recorded. Corneal astigmatic measurements were converted into vector components—J0 and J45. Intra-device repeatability and agreements of measurements amongst the devices were evaluated using repeatability coefficients (RCs) and Bland–Altman plots, respectively. All devices demonstrated comparable repeatability for Km (p ≥ 0.138). ANTERION had the lowest RC for J0 amongst the devices (p ≤ 0.039). Systematic difference was found for the Km and J0 obtained with IOLMaster500 compared to either SSOCTs (p ≤ 0.010). The ACD and AL measured by IOLMaster500 showed a higher RC compared with either SSOCTs (p < 0.002). Systematic difference was found in CCT and ACD between the two SSOCTs (p < 0.001), and in AL between ANTERION and IOLMaster500 (p < 0.001), with a mean difference of 1.6 µm, 0.022 mm and 0.021 mm, respectively. Both SSOCTs demonstrated smaller test–retest variability for measuring ACD and AL compared with IOLMaster500. There were significant disagreement in keratometry and AL measurements between the SSOCTs and PCI-based device; their measurements should not be considered as interchangeable.

## Introduction

The advancement of surgical techniques and intraocular lens (IOL) design has greatly improved the outcomes of cataract surgery but also raised patients’ expectations for refractive-error free vision. Accurate measurements of axial length (AL), corneal power, astigmatic axis and other anterior segment parameters are crucial for IOL power calculation^1^, especially with the growing popularity of toric IOL implantation to correct preexisting corneal astigmatism at the time of cataract surgery. The IOLMaster (Carl Zeiss Meditec, Jena, Germany) was the first commercially available optical biometry device and has been used widely for IOL power calculation^2,3^. The IOLMaster500 is based on the principle of partial coherence interferometry (PCI) and have demonstrated accurate measurement for IOL power calculation in both routine and complicated cataract cases^4,5^.

Anterior segment ocular coherence tomography (OCT) has the advantage of high tissue penetration, high scanning speed and has the ability to identify both anterior and posterior surfaces with high repeatability^6^. OCT is emerging as a new modality for corneal tomographic analysis^7^. The development of swept-source OCT allows quick, one-station, detailed imaging of the cornea, the anterior chamber, as well as the anterior and posterior surfaces of the lens with improved image resolution and scan speed^8,9^. Since the first generation CASIA SS-1000 (Tomey, Nagoya, Japan), the technology continues to improve. For instance, compared with CASIA SS-1000, the new CASIAII offers a faster scan-speed (50,000 vs. 30,000 A-scans/s) and a higher transverse resolution (800 A-scans/B-scan vs. 256 A-scans/B-scan) for 360° imaging of the anterior chamber angle using 18 evenly-spaced radial scans over 36 angle locations. The CASIAII (introduced in 2016–2017) and the ANTERION (introduced in 2019, Heidelberg Engineering, Heidelberg, Germany) are two swept-source OCTs that were developed recently.

The purpose of this study was to compare the repeatability and agreement between ANTERION, CASIAII and IOLMaster500 for measurements of keratometry and anterior chamber depth (ACD). Central corneal thickness (CCT) measurements obtained from ANTERION and CASIAII were compared. Axial length measurements obtained from IOLMaster500 and ANTERION were also compared.

## Methods

This was a cross-sectional study conducted at The Chinese University of Hong Kong, Department of Ophthalmology and Visual Science between June and September 2019. Eyes that had previous ocular surgery (including corneal refractive surgery and lens extraction) were excluded. Apart from cataract, eyes with ocular disease, including corneal pathologies (e.g. cornea ectasia, pterygium), infectious disease (e.g. infective keratitis, viral conjunctivitis), and/or problems with dry eyes were excluded. Patients with myopia of more than − 6.0 D and visual acuity of worse than Snellen 6/12 were also excluded. Written informed consents were obtained from all subjects. The study was conducted in accordance with the ethical standards stated in the 2013 Declaration of Helsinki and approved by Hong Kong Kowloon Central Research Ethics Committee with written informed consent obtained.

### IOLMaster500

The IOLMaster500 (Carl Zeiss Meditec, Jena, Germany) utilizes the principle of PCI to measure the AL. It evaluates the keratometry with a six-point telecentric technique and an image-based slit lamp system for ACD measurements. It does not provide lens thickness nor CCT measurement. It measures AL from the anterior corneal surface to the retinal pigmented epithelium along the line of sight.

### CASIAII swept-source OCT

The CASIAII swept-source OCT (Tomey, Nagoya, Japan) is a form of Fourier-domain OCT that utilizes a swept-source wavelength of 1310 nm as the light source and a photodetector to detect wavelength-resolved interference signal^9^, with improved in image resolution, scan speed, width, and depth^8,9^. At a scan speed of 50,000 A scan/s and an axial resolution of < 10 µm, it allows multiple high-resolution, up to 256 cross-sectional images of the entire anterior segment and angle to be captured within 3 s. The maximum scan width is 16 mm with a scan depth of 13 mm^10^. It also provides corneal topography and automatic measurement software. With the measuring mode of corneal map and lens biometry, 16 radial cross-sectional images, with 800 A-scan per line sampling, a scan width of 16 mm and a scan depth of 11 mm can be delivered with a scan speed of 0.3 s. It only images the anterior segment with no AL measurement.

### ANTERION swept-source OCT

The ANTERION swept-source OCT (Heidelberg Engineering, Heidelberg, Germany) is another form of Fourier-domain OCT that offers a fast scan-speed of 50,000 A-sans/second. It utilizes a 1300 nm light source to offer an axial resolution of < 10 µm. Compared with the CASIAII, it provides a wider scan width (up to 16.5 mm wide) and a deeper scan depth range (14 ± 0.5 mm)^11^. This allows visualization of detail corneal, anterior chamber, angle, and lens (both anterior and posterior surfaces). The four different in-built imaging Apps—Cornea App, Cataract App, Metrics App, and Imaging App—allows a comprehensive examination of the anterior segment imaging, corneal topography and tomography, anterior segment biometry, IOL calculation, and AL measurement in a single scan. With the ANTERION Cornea App, 65 radial B-scan images (256 A-scans per B-scan) are acquired in less than 1 s and the data can generate corneal maps of 8 mm in diameter^12^. It measures the distance between the anterior corneal surface and the retinal pigment epithelium, along the line of sight, as the AL^12^.

### Imaging and measurements

One randomly selected eye of each subject was imaged by all three instruments. Each eye was scanned three times for each instrument to obtain clear images of the anterior and posterior corneal surfaces. The sequence of measurement recording between the ANTERION, CASIAII, and IOLMaster500 was not fixed. The time elapsed between measurement devices included a short break for the patient to relax for tear film recovery and avoid fatigue. All measurements were performed by a single experienced technician (G.L.) and were taken under dim room illumination. Patients were asked to blink in between consecutive scans to produce an optically smooth tear film, thereby improving the reflectivity of the cornea. During the imaging, the subjects were asked to fixate at an internal fixation target. To avoid lid artifact, the technician would retract the upper and lower lids of the participant while taking the imaging. To ensure stable corneal conditions, patients were asked to withhold soft contact lens wear for 2 weeks before the evaluation; none of our patients wore hard contact lens.

Power vector analysis was conducted for obtaining vectors along the 0° and 45° meridians according to the following equation: J0 = (− [Ksteep − Kflat]/2 cos 2α), and J45 = (− [Ksteep − Kflat]/2 sin 2 α), for comparison in a Cartesian coordinate system^13^. Ksteep, Kflat, and α represent the steep keratometry, flat keratometry, and axes values, respectively. J0 represents the astigmatic component along the vertical meridian (with-the-rule or against-the-rule astigmatism), while J45 represents oblique astigmatism. Fellow eye data were flipped to avoid neutralization of the J45 vector component when both eyes data were included for comparison.

R 3.2.5 (R Foundation, Vienna, Austria) was used for statistical analysis. Repeatability coefficients (RCs) was used to evaluate the repeatability of measurements obtained by the IOLMaster500, CASIAII and ANTERION. RC is defined as the 95% confidence limit of the difference of measurement between examinations, which is equal to 1.96 $\sqrt{\left(2\times \mathrm{pooled test}-\mathrm{retest variance}\right)}$. A high RC value represents a low test–retest variability, and vice versa^14^.

Comparison of RCs for parameters between the CASIAII vs ANTERION, ANTERION vs IOLMaster500, and CASIAII vs IOLMaster500 were evaluated by empirical bootstrap resampling with 2000 replicates. The first attempted measure of each subject is evaluated. Bland–Altman plots were used to assess the agreement between measurements of the two devices. Differences between the measurement values were plotted against the mean values of the measurements, and the 95% limits of agreement (LoA), which is equal to the mean difference ± 1.96 × SD, were evaluated. Systematic differences of each parameter between the two devices were compared using the t-test. Proportional bias was investigated by linear regression of the difference in values measured by the two devices.

Based on test for equality of two within-subject variances in a parallel design, a sample of size 45/46/45 is needed to detect a difference in variance in terms of ratio of 1.6/1.8/2 at 5% level of significance with power of 60%/80%/90%^15^. A *p* value less than 0.05 was considered statistically significant. False discovery rate, which measures the percentage of false discovery due to random error, was evaluated for multiple statistical tests with the threshold of p-value < 0.05 ^16^.

## Results

A total of 48 eyes of 48 patients were included. The mean age was 57.6 ± 14.8 years. Table 1 summarizes the repeatability outcomes of the parameters obtained from ANTERION, CASIAII, and IOLMaster500. These include the mean keratometry (Km), J0 and J45 vector components of astigmatism, ACD (measured from epithelium to anterior lens surface), CCT (for the two OCT devices only), and AL (for ANTERION and IOLMaster500 only). Both ANTERION and CASIAII demonstrated comparable test–retest repeatability for measurements of Km and CCT (p ≥ 0.138). The J0 vector component of astigmatism measured by CASIAII showed a significantly greater RC (p = 0.002) compared with the J0 measured by the ANTERION. The repeatability of the J45 vector component of astigmatism were similar across all the devices (p ≥ 0.072). RCs were greater for the measurements obtained from IOLMaster500 compare with those obtained by either OCTs although significant differences were found only in ACD and AL measurements (p ≤ 0.002).

**Table 1:** Repeatability outcomes for biometric measurements obtained using the ANTERION, CASIA II and IOLMaster500.

Parameters	Devices	Mean (± SD)	RC (95% CI)	p-value for pairwise comparison in RC		
				ANTERION vs CASIAII	ANTERION vs IOLMaster500	CASIAII vs IOLMaster500
Km (D)	ANTERION	43.878 ± 1.415	0.2345 (0.2010–0.2681)	0.600	0.138	0.254
	CASIA II	43.851 ± 1.440	0.2484 (0.2125–0.2843)			
	IOLMaster500	43.902 ± 1.456	0.3148 (0.2666–0.3629)			
J0 (D)	ANTERION	0.305 ± 0.457	0.2003 (0.1716–0.2289)	0.002	0.039	0.361
	CASIA II	0.376 ± 0.422	0.3217 (0.2752–0.3682)			
	IOLMaster500	0.207 ± 0.469	0.5448 (0.4614–0.6282)			
J45 (D)	ANTERION	− 0.002 ± 0.180	0.2232 (0.1913–0.2551)	0.980	0.245	0.072
	CASIA II	0.029 ± 0.196	0.2115 (0.1809–0.2420)			
	IOLMaster500	− 0.024 ± 0.226	0.3336 (0.2825–0.3846)			
ACD (mm)	ANTERION	2.899 ± 0.597	0.0311 (0.0264–0.0352)	0.216	< 0.001	< 0.001
	CASIA II	2.912 ± 0.586	0.0260 (0.0227–0.0298)			
	IOLMaster500	2.932 ± 0.563	0.1947 (0.1652–0.2241)			
CCT (µm)	ANTERION	543.17 ± 32.20	2.1759 (1.8648–2.4869)	0.575	–	–
	CASIA II	543.02 ± 33.45	2.3223 (1.9868–2.6579)			
AL (mm)	ANTERION	23.683 ± 1.137	0.0154 (0.0130–0.0178)	–	0.002	–
	IOLMaster500	23.881 ± 1.323	0.0260 (0.0221–0.0299)			

*Km* mean keratometry, *J0* J0 vector components of astigmatism, *J45* J45 vector components of astigmatism, *ACD* anterior chamber depth, *AL* axial length, *CCT* central corneal thickness, *RC* repeatability coefficient.

10.9% of the 5 significant results out of the 14 tests are expected to be false discoveries due to random error as determined by the false discovery rate.

**Figure 1:**
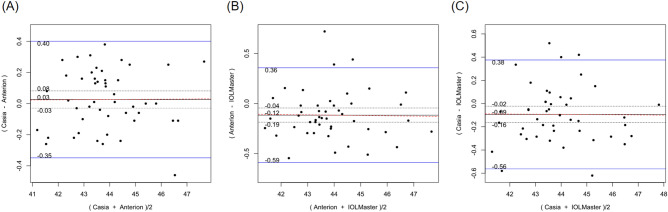
Bland–Altman plots showing the pairwise agreement between ANTERION vs CASIAII (**A**), ANTERION vs IOLMaster500 (**B**), and CASIAII vs IOLMaster500 (**C**) for mean keratometry (Km).

**Figure 2:**
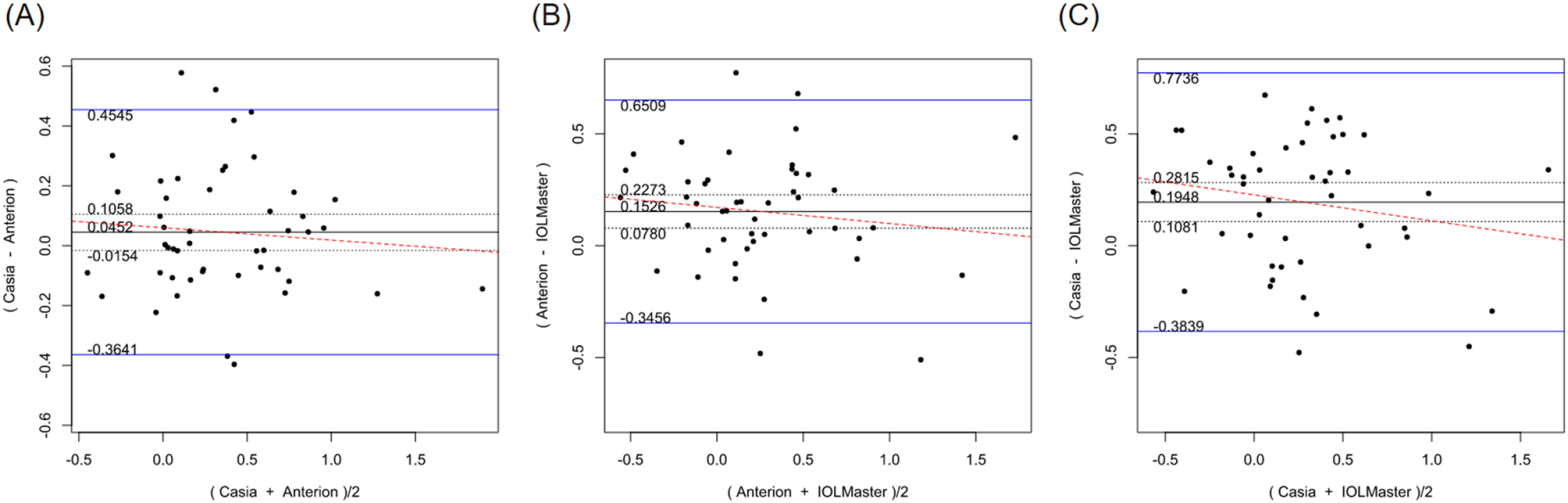
Bland–Altman plots showing the pairwise agreement between ANTERION vs CASIAII (**A**), ANTERION vs IOLMaster500 (**B**), and CASIAII vs IOLMaster500 (**C**) for J0 vector component of astigmatism.

**Figure 3:**
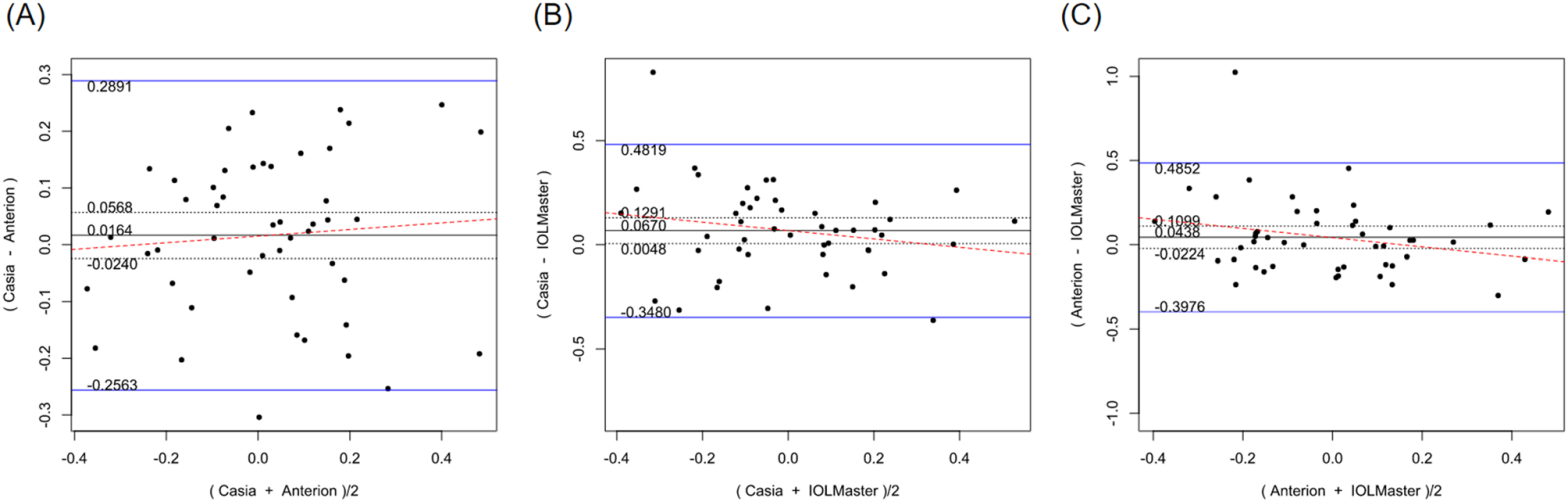
Bland–Altman plots showing the pairwise agreement between ANTERION vs CASIAII (**A**), ANTERION vs IOLMaster500 (**B**), and CASIAII vs IOLMaster500 (**C**) for J45 vector component of astigmatism.

**Figure 4:**
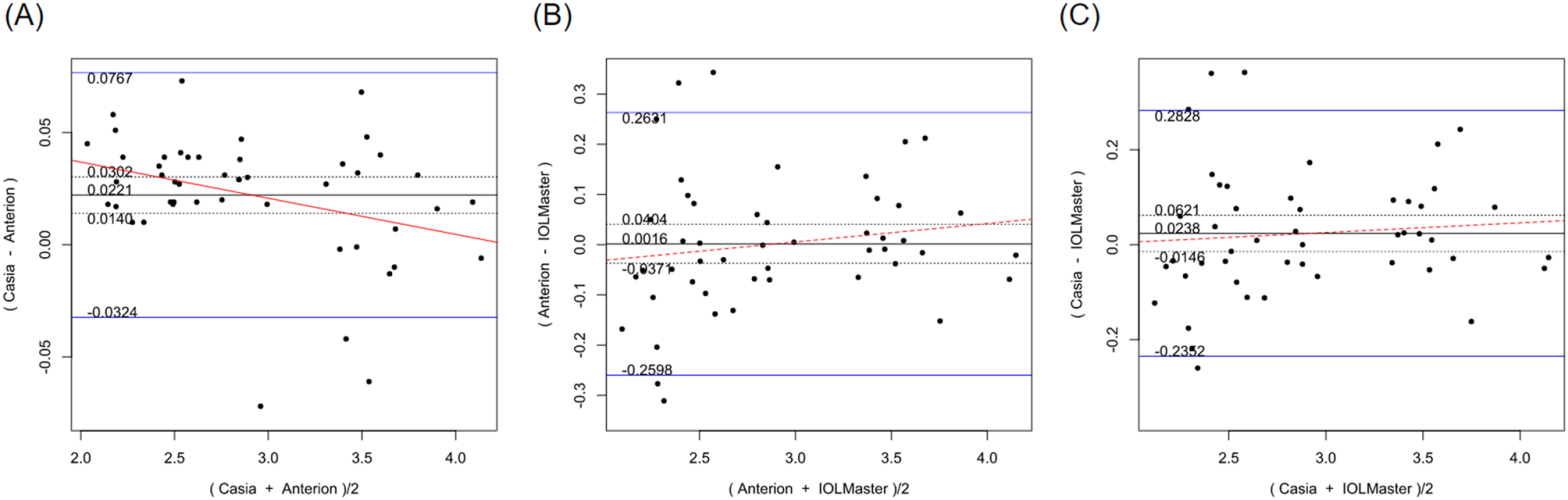
Bland–Altman plots showing the pairwise agreement between ANTERION vs CASIAII (**A**), ANTERION vs IOLMaster500 (**B**), and CASIAII vs IOLMaster500 (**C**) for anterior chamber depth (ACD).

**Figure 5:**
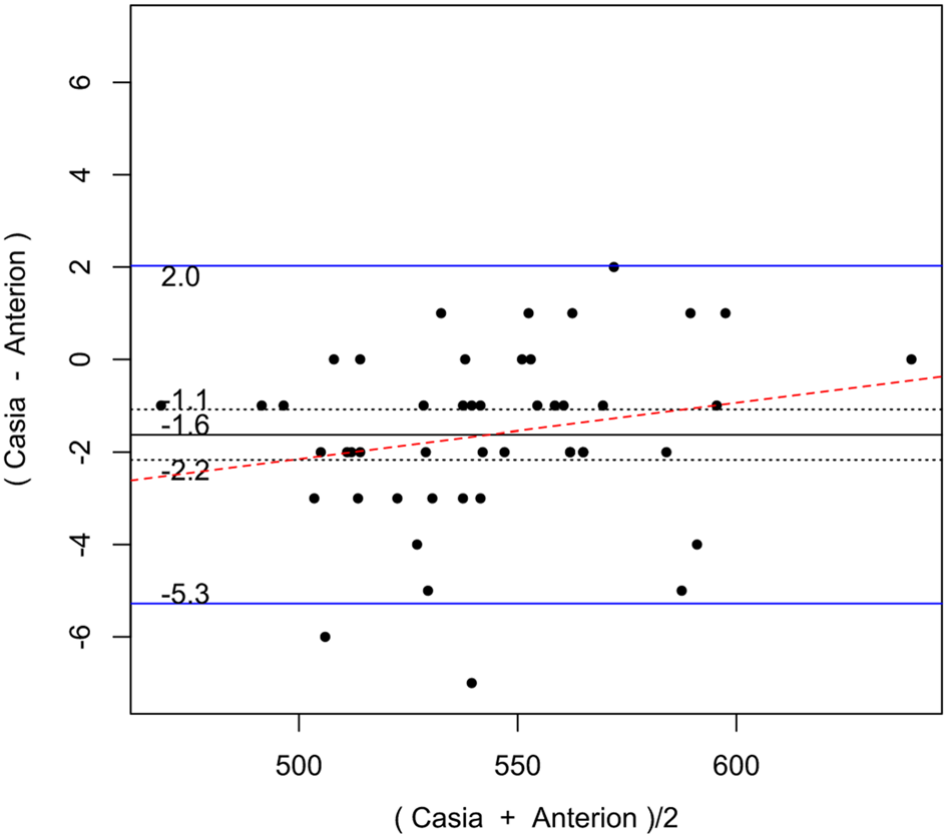
Bland–Altman plots showing the pairwise agreement between ANTERION vs CASIAII for central corneal thickness (CCT).

**Figure 6:**
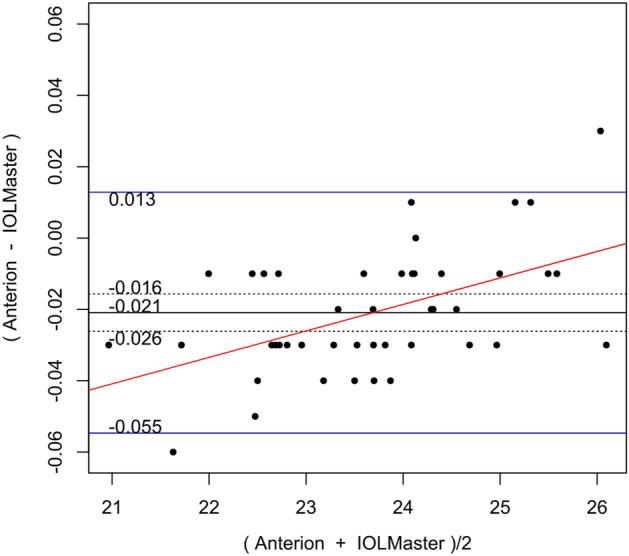
Bland–Altman plots showing the pairwise agreement between ANTERION vs IOLMaster500 for axial length (AL).

Table 2 shows the systematic differences and the 95% LoA between ANTERION versus CASIAII, ANTERION versus IOLMaster500, and CASIAII versus IOLMaster500 measurements. Systematic differences were found in CCT and ACD between ANTERION and CASIAII (p < 0.001). However, only the ACD measurement showed a proportional bias (p = 0.018) and not the CCT measurement (p = 0.149). There were systematic differences for the Km and the J0 vector component between either OCT devices and the IOLMaster500. The mean difference of Km between ANTERION and IOLMaster500 was − 0.115 D (p = 0.002) (span of 95% LoA was 0.945 D [range: − 0.588 to 0.357 D]; maximum absolute 95% LoA was 0.588 D) and the mean differences of J0 was 0.153 D (p < 0.001) (span of 95% of LoA was 0.997 D [range: − 0.346 to 0.651 D]; maximum absolute 95% LoA was 0.651 D). There was no proportional bias between the two devices (p ≥ 0.104). The mean differences of Km, J0 and J45 vectors of astigmatism between CASIAII and IOLMaster500 were − 0.093 D (p = 0.010) (span of 95% of LoA was 0.908 D [range: − 0.532 to 0.376 D]; maximum absolute 95% LoA was 0.532 D), 0.195 D (p < 0.001) (span of 95% of LoA was 1.158 D [range: − 0.384 to 0.774 D]; maximum absolute 95% LoA was 0.774 D), and 0.067 D (p = 0.035) (span of 95% of LoA was 0.830 D [− 0.348 to 0.482 D]; maximum absolute 95% LoA was 0.482 D), respectively. None of these comparisons showed any proportional bias between the two devices (p ≥ 0.185). As for the AL, which was only measured by ANTERION and IOLMaster500, there was a significant mean difference of − 0.021 mm (p < 0.001) (span of 95% of LoA was 0.068 mm [range: − 0.055 to 0.013 mm]; maximum absolute 95% LoA was 0.055 mm) between the two devices; a proportional bias was observed (p < 0.001). The Bland–Altman plots of the parameters measured by the three devices were shown in Figs. 1, 2, 3, 4, 5, and 6.

**Table 2:** Systematic differences and proportional biases between different devices.

Parameters	Systematic difference			Proportional bias		
	Mean differences	95% LoA	p	Scaling difference	R^2^	p
**CASIA II vs ANTERION**						
Km (D)	0.026	− 0.349 to 0.401	0.351	0.001	7.33 × 10^–5^	0.954
J0 (D)	0.045	− 0.364 to 0.455	0.140	− 0.041	0.008	0.557
J45 (D)	0.016	− 0.256 to 0.289	0.419	0.058	0.007	0.581
ACD (mm)	0.022	− 0.032 to 0.077	< 0.001	− 0.016	0.116	0.018
CCT (µm)	− 1.625	− 5.278 to 2.028	< 0.001	0.012	0.045	0.149
**ANTERION versus IOLMaster500**						
Km (D)	− 0.115	− 0.588 to 0.357	0.002	− 0.003	3.41 × 10^–4^	0.902
J0 (D)	0.153	− 0.346 to 0.651	< 0.001	− 0.072	0.018	0.374
J45 (D)	0.044	− 0.398 to 0.485	0.189	− 0.274	0.058	0.104
ACD (mm)	0.002	− 0.260 to 0.263	0.932	0.037	0.026	0.278
AL (mm)	− 0.021	− 0.055 to 0.013	< 0.001	0.007	0.260	< 0.001
**CASIA II versus IOLMaster500**						
Km (D)	− 0.093	− 0.562 to 0.376	0.010	− 0.002	9.55 × 10^–5^	0.948
J0 (D)	0.195	− 0.384 to 0.774	< 0.001	− 0.116	0.032	0.232
J45 (D)	0.067	− 0.348 to 0.482	0.035	− 0.201	0.039	0.185
ACD (mm)	0.024	− 0.235 to 0.283	0.219	0.020	0.008	0.547

*Km* mean keratometry, *J0* J0 vector components of astigmatism, *J45* J45 vector components of astigmatism, *ACD* anterior chamber depth, *CCT* central corneal thickness, *95% LoA* 95% limit of agreement.

9.8% of the 10 significant results out of the 28 tests are expected to be false discoveries due to random error as determined by the false discovery rate.

## Discussion

Our study compared the two anterior segment swept-source OCT devices (CASIAII and ANTERION) with the widely used, PCI-based IOLMaster500 for measurements of the corneal topography, ACD, and AL. Previously, we had also compared the performance of CASIAII and ANTERION for the measurement of the angle parameters^17^. Other relevant studies that were recently published included comparison between ANTERION and IOLMaster 500^18,19^, Tomey CASIA SS-1000 and ANTERION^20,21^, IOLMaster700 and CASIAII^22^, and amongst three swept-source OCT devices (CASIAII, ANTERION, and IOLMaster700)^23^.

In the current study, comparison between ANTERION and IOLMaster500 showed that the swept-source OCT had superior repeatability in measuring AL. There was also a significant systematic difference and proportional bias between ANTERION and IOLMaster500 for AL measurements. AL measured by the ANTERION were shorter compared to that measured by the IOLMaster500 although a mean difference of 0.021 mm may not be clinically significant given that a difference in AL of 0.030 mm would only result in a spherical error of approximately 0.1 D in eyes with average AL and corneal curvature^1^. This finding correlates with the recent publication that compared ANTERION and IOLMaster500^18^. The new IOLMaster700 (Carl Zeiss Meditec, Jena, Germany) also bases on swept-source OCT to measure AL. Some studies found no difference for AL measurements between the IOLMaster500 and 700^24,25^, whereas others reported significant difference between the two devices^26,27^. Recent study that compared ANTERION and IOLMaster 700 (both are swept-source OCT based devices) showed excellent agreement in measuring AL^23^. Swept-source OCT device had demonstrated a superior ability to perform measurement compared with PCI-based device in the case of dense cataract and posterior subcapsular cataract^28^. The ability to use a longer wavelength than that used by PCI (780 nm) can reduce light scattering from opaque media, allowing greater penetration through a severe cataract. However, our patients had an axial length in the normal range and a relatively good vision (at least 6/12), the performance of these devices for eyes with extreme AL and dense cataract requires further study.

The ACD obtained with the ANTERION and CASIAII showed similar repeatability. There was a mean difference of 0.022 mm with a span of 95% LoA of 0.109 mm and a maximum absolute 95% LoA of 0.077 mm. The ACD measurements obtained with the ANTERION were shorter than CASIAII. Such difference may not affect IOL power calculation because a difference of 0.100 mm in ACD would only lead to an approximately 0.15 D change in refraction in eyes with an average AL and corneal curvature^1^. Either swept-source OCTs device provided more repeatable measurements of ACD compared to the IOLMaster500. There is no statistically significant systematic difference or proportional bias between the ACD of IOLMaster500 compared with either OCT. However, the span of 95% LoA were as much as 0.5 mm (and a maximum absolute 95% LoA of 0.263 mm) when compared with either ANTERION and CASIAII, and this can be clinically significant for IOL power calculation. Comparison between IOLMaster500 and a different swept-source OCT biometer (OA-2000, Tomey, Nagoya, Japan) also found a wide span of 95% LoA (0.41 mm) in ACD measurements^29^. Unlike the OCTs that measure the ACD from OCT image that is aligned with the optical axis of the eye, the principle of IOLMaster500 for measuring ACD is based on an optical section through the anterior chamber using a lateral slit beam illumination technique, which can be prone to operator errors^26^.

Although CCT measurement is not crucial for IOL power calculation, it is important for eyes planning for corneal refractive surgery. The CCT obtained with the two swept-source OCTs demonstrated similar repeatability but a significant systematic difference of 1.6 µm with a span of 95% LoA of 7.3 µm (and a maximum absolute 95% LoA of 5.3 µm). A much wider span of 95% LoA of 35 µm has been found between two other swept-source OCT-based biometers, IOLMaster 700 and ARGOS (Movu, Komaki, Japan)^30^. The reason for the differences could be the inconsistent measurement location and different measurement strategies between the different swept-source OCTs.

The Km measurements demonstrated comparable repeatability for all three devices. However, the Km measured by IOLMaster500 showed a systematic difference when compared with either ANTERION or CASIAII, implying between-device disagreement. The Km measured by IOLMaster500 was significantly steeper than ANTERION (0.115 D) and CASIAII (0.093 D). It has been suggested that a Km difference of 0.20 D between the devices is sufficient to give different optimized constants for IOL power calculation^31^. Intraocular lens constant optimization based on the difference in magnitude in Km values has been proposed. Karunaratne et al. demonstrated that the keratometry readings of the IOLMaster and Pentacam (Oculus, Wetzlar, Germany) were not equivalent, but their keratometry readings were similarly effective in IOL power calculations after constant optimization^32^. Similarly, although our findings showed that the Km measured by IOLMaster500 significantly differs from either swept-source OCTs, such disagreement could be refined by readjustment of intraocular lens constant in clinical practice.

As for the J0 and J45 astigmatic vector measurements, the J0 measured by ANTERION demonstrated a significantly lower value of RC compared with CASIAII, and a similar RC for J45. Although the RC of J0 and J45 obtained with IOLMaster500 did not show a significant difference compared with either OCT (apart from a significantly higher RC for J0 when compared with the ANTERION), there is a trend of higher RC values for the vector components obtained with the IOLMaster500. The between-instrument agreement of IOLMaster500 and either OCT for the astigmatic vector measurements was poor. There was a systematic difference for the J0 obtained with the IOLMaster500 compared with either the ANTERION or CASIAII. A systematic difference was also found for the J45 between the IOLMaster500 and CASIAII. These observations are clinically important, given that the accuracy of astigmatism measurements is crucial for the implantation of toric IOL. Our data suggested that ANTERION demonstrated superior repeatability for the measurement of J0, which represents with-the-rule and against-the-rule astigmatism, and its agreement with the IOLMaster500 was poor. In practice, it is important to perform corneal topography when before implanting a toric IOL in cataract surgery since irregular corneal astigmatism, which is not correctable by toric IOL, has been shown to increase with increasing age^33^. It is likely that the difference between PCI-based and swept-source OCT-based devices exists because of the different methods used to analyze the mires of spots reflected from the cornea. The IOLMaster500 measures keratometry from the anterior cornea in the 2.5 mm zone using only 6 spots of light projected on the cornea^24^ is different from swept-source OCTs that utilizes multiple evenly spaced radial B-scans for the measurement^24^. Therefore, the astigmatic axis measured by the swept-source OCT and the PCI-based IOLMaster500 should not be considered interchangeable. It remains to be elucidated how the differences of the measurements would affect the predicted residual astigmatism after toric IOL implantation.

This study has several limitations. We did not grade the severity of cataract in our patients. A previous study showed that swept-source OCT-based device was more effective in obtaining biometric measurements in eyes with posterior subcapsular and dense nuclear cataract^26^. Our study did not recruit patients with visual acuity worse than Snellen 6/12. Our study did not investigate the refractive outcome after cataract surgery; we, therefore, could not determine the refractive prediction errors because of the measurement differences in IOL power calculation. Further study is required to verify how the findings in the current study that may affect the cataract surgical outcome in clinical practice.

In conclusion, both ANTERION and CASIAII demonstrated excellent repeatability in biometry and corneal measurements. Favorable agreement was demonstrated in keratometry and its astigmatic axis measurements between the two swept-source OCTs. On the other hand, IOLMaster500 demonstrated lower repeatability for ACD and AL measurements. There was significant disagreement in keratometry and AL measurements between swept-source OCT and PCI-based devices.
